# Supplementary material for: Policy and market forces delay real estate price declines on the US coast
Source: Nat Commun. 2024 Mar 12;15:2209. doi: 10.1038/s41467-024-46548-6 (PMC10928214; doi:10.1038/s41467-024-46548-6)
Supplement: Supplementary file 1 — Supplementary Information [file 41467_2024_46548_MOESM1_ESM.pdf]

Supplementary Information For "Policy and Market Forces Delay Real Estate Price Declines on the US Coast"

Dylan E. McNamara[1], Martin D. Smith[2,*], Zachary Williams[1,2], Satjya Gopalakrishnan[3], and Craig E. Landry[4]

[1]Department of Physics and Physical Oceanography and Center for Marine Science, University of North Carolina, Wilmington, NC
[2]Nicholas School of the Environment and Department of Economics, Duke University, Durham, NC
[3]Department of Agricultural, Environmental, and Development Economics, The Ohio State University, Columbus, OH
[4]Department of Agricultural and Applied Economics, University of Georgia, Athens, GA
[*]martin.smith@duke.edu

Contents

1 Additional Figures 1
2 Model Features and Parameterization 5
3 Rationale for Scenarios 9
4 Sensitivity Analysis 10
5 Additional Scenarios 20
References 23

1 Additional Figures

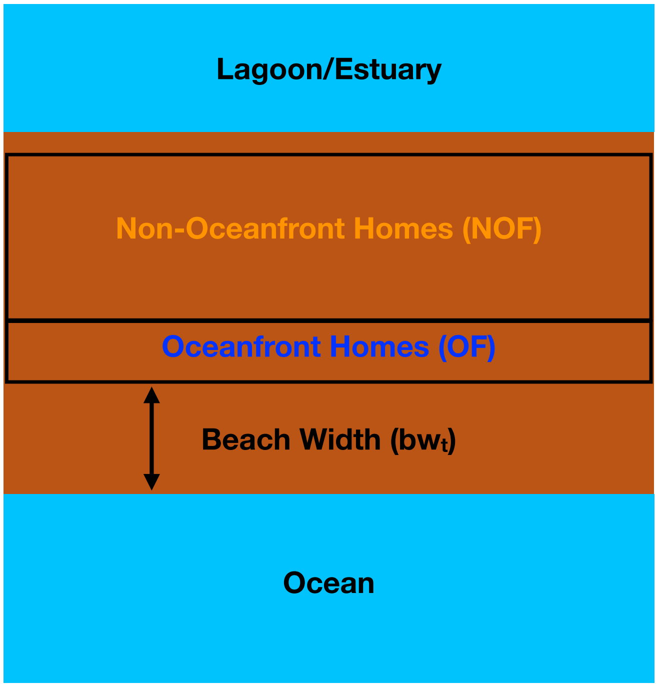

Supplemental Figure 1. Model Domain Schematic. Plan view orientation of oceanfront and non-oceanfront property markets with respect to ocean and lagoon/estuary. Beach width is indicated as the horizontal distance that spans the zone between the ocean and oceanfront homes.

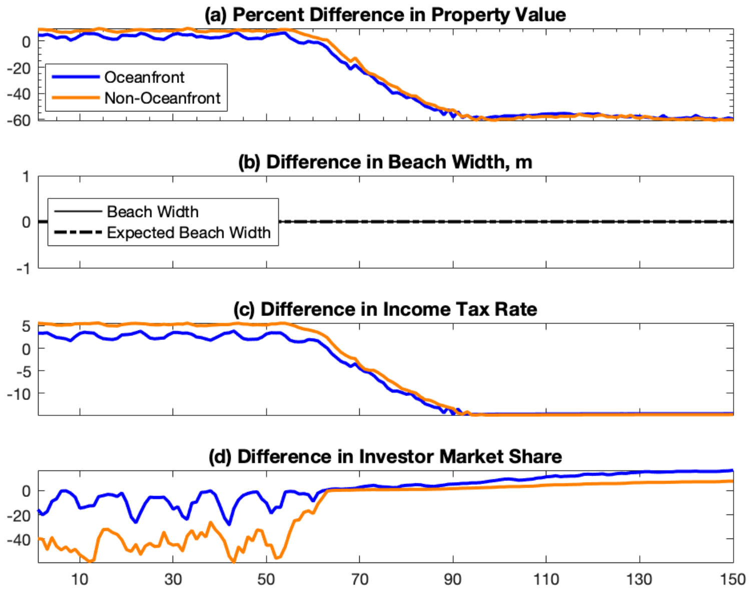

Supplemental Figure 2. No Agent Flux. Model results for scenario where agent flux is turned off. Results displayed as differences compared to baseline scenario.

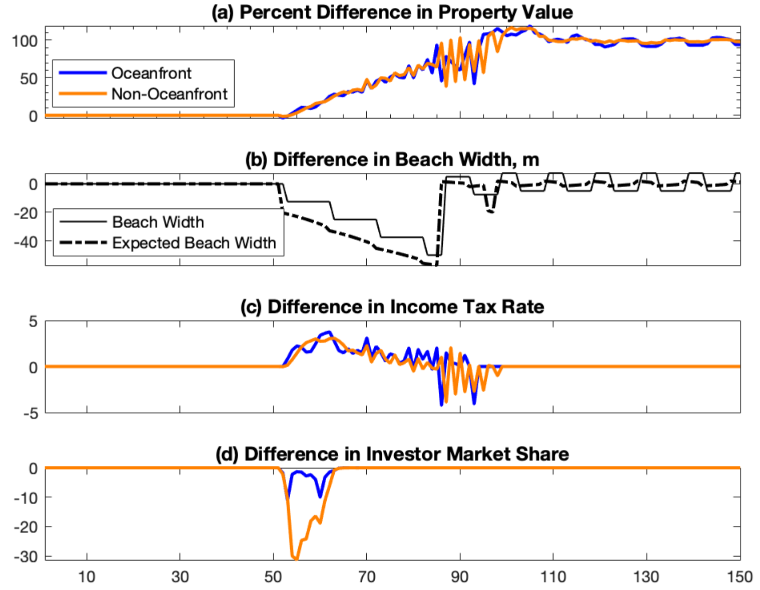

Supplemental Figure 3. Simultaneous Increase in Outside Markets and Reduction in Nourishment Subsidy. Model results for scenario where outside property markets increase in value and the nourishment subsidy is reduced. Results displayed as differences compared to baseline scenario.

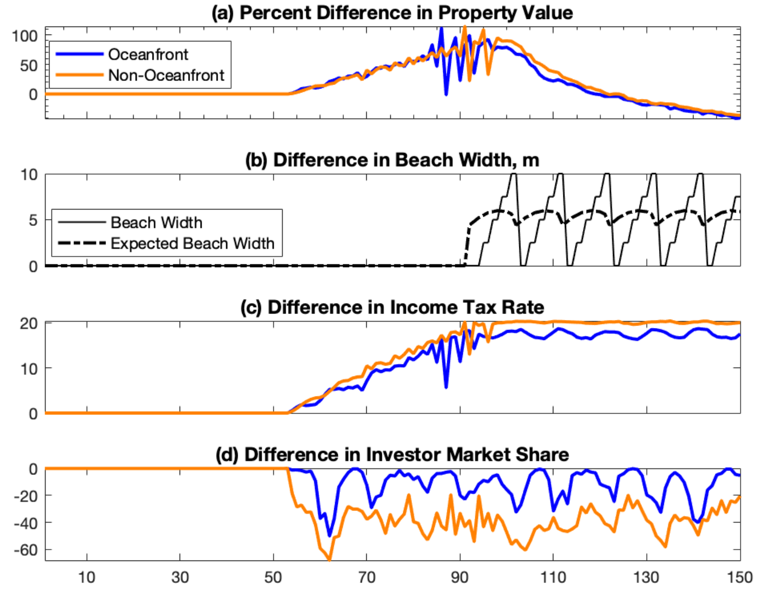

Supplemental Figure 4. Comparison of the Offsetting Effects of SLR and Appreciation in Outside Markets to No SLR and Stable Outside Markets. Model results compare two scenarios that each differ from the baseline scenario. The first is with outside markets that increase along with SLR, which allows for the possibility that market appreciation compensates for SLR. The second is with stable outside markets and but no SLR. Results displayed are differences between the two scenarios.

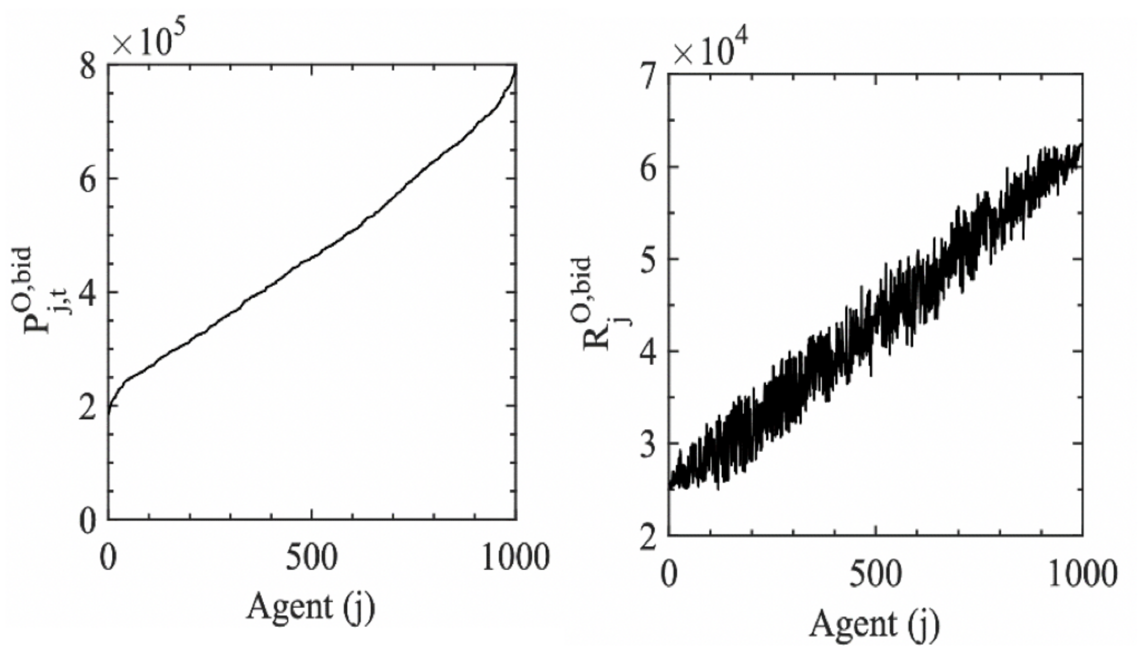

Supplemental Figure 5. Example of price and rent bids for prospective owners.

2 Model Features and Parameterization

Table 1. Model parameters

Parameter	Definition	Value	Justification
δ	Discount Rate	0.06	See Note 1 below
τ^p	Property tax rate	0.01	See Note 2 below
τ^{inc}	Income tax rate	[0.10 0.37]	See Note 3 below
γ	Depreciation rate	0.01	See Note 4 below
HV	Housing services	0	See Note 5 below
m	Management fee	\$2000 year^{-1}	See Note 6 below
$\overline{r^p}$	Real estate risk premium	0.005	See Note 7 below
r^{OF}	Fixed OF add. risk	0.02	See Note 8 below
a_2	SL risk	0.70 m^{-1}	See Note 9 below
n	SL risk exponent	2	See Note 9 below
h^{elev}	Property elevation	1m	See Note 10 below
a_1	Storm return scaling	0.10 years	See Note 11 below
λ_t	Storm return time	20 years	Keim et al., 2007[1]
π_j	Agent risk multiplier	[0.5 1.5]	See Note 12 below
WTP_j^{base}	Base WTP	[23500 40000]\$	See Note 13 below
β^{OF}	Hedonic width exponent OF	0.2	See Note 14 below
β^{NOF}	Hedonic width exponent NOF	0.1	See Note 15 below
α_j	Hedonic scaling	[9000 12000] \$ m$^{-\beta}$	See Note 16 below
t_{bw}	Beach width averaging time	30 years	See Note 17 below
P_e^{OF}	External market price OF	\$500,000	See Note 18 below
P_e^{NOF}	External market price NOF	\$400,000	See Note 18 below
h	Bubble coefficient	1x10^{-6}\$$^{-1}$	See Note 19 below
ϕ	Flux parameter	2x10^{-6}	See Note 20 below
ρ	Nourishment tax ratio	3	See Note 21 below
f	Fixed cost of nourishment	\$1,000,000	See Note 22 below
bw_o	Nourished beach width	70m	See Note 22 below
L	Alongshore extent of nourishment	15km	See Note 22 below
D	Shoreface depth	20m	See Note 22 below
c	Sand cost	\$10 per m^3	See note 22 below
ψ_{bw}	Yearly erosion rate	1.25 m	Hapke et al., 2011[2]
ψ_{slr}	Yearly rate of sea level rise	.01 m	See Note 23 below

Note 1: The discount rate in this context is the mortgage interest rate. The rate 6% is used in the beach nourishment simulations in Gopalakrishnan et al., 2011[3]. The average 30-year nominal mortgage rate from 1971-2023 is 7.74% based on 30-Year Fixed Rate Mortgage Average in the United States, Percent, Weekly, Not Seasonally Adjusted. The average rate of inflation in this time is 4%, suggesting a real mortgage rate close to 3.75%. We use 3% and 9% for low and high values in the sensitivity analysis below.

Note 2: Base tax rates for Dare County, NC (not including nourishment) are in the range of 0.0075, depending on the municipality.
https://www.darenc.gov/departments/tax-department/tax-rates
Base tax rates for New Hanover County, NC are in the range of 0.0075 for Carolina Beach and Kure Beach, whereas for the City of Wilmington the rate is 0.009175
https://www.nhcgov.com/CivicAlerts.aspx?AID=425#:~:text=City%20of%20Wilmington%20Municipal%20Services,Beach%20%E2%80%93%2026.58%20cents%20per%20%24100
The value 1% rounds up but is still below rates for inland cities in North Carolina, e.g. 1.3% for the City of Durham, NC.
https://www.dconc.gov/county-departments/departments-f-z/tax-administration/tax-rates

Note 3: This is the range of federal marginal income tax rates.

https://www.irs.gov/newsroom/irs-provides-tax-inflation-adjustments-for-tax-year-2024

Note 4: There is considerable heterogeneity in estimates of housing depreciation with evidence that depreciation rate varies across the life of the property, differs across regions, and varies by renter versus owner-occupied. Many estimates fall in the range of 0.5%-2%[4,5]. The sensitivity analysis uses a low value of 0% and a high value of 2%. Because housing depreciation and base risk premium are added together in the denominator of the user cost model, sensitivity one or the other is equivalent. See notes 7-8 below.

Note 5: A component of WTP is dependent on beach width, but the base amount is not. Because HV and WTP are added together in the numerator, their influence cannot be separately identified. We thus fix $HV = 0$ and vary base WTP to explore implications.

Note 6: Management fees can be flat fees or a percentage of rental income, often ranging from 5-10%. A $400,000 property could rent for $40,000/year, so 5% would be a $2,000 management fee. Real estate websites provide some examples, but numbers from the academic literature are difficult to find, and the Federal Reserve does not track them.
https://www.thebalancemoney.com/a-breakdown-of-property-management-fees-4589926
https://www.rentspree.com/blog/how-much-do-property-management-companies-charge
https://www.rpmraleigh.com/property-management-pricing

Note 7: Using asset-pricing approach, Cannon, Miller, and Pandher, 2006[6] find that housing investment exhibits a positive return to risk. The average 10-yr T-bill rate for 1987-present is 4.6%. The nominal rate of increase in the housing price index is 4.4% over this period, or 0.2% below the risk-free rate. The actual rate of return on residential housing investments would include the rental income net of management costs, depreciation, and debt service. Given the slow rate of capital growth, we choose a low risk premium of 0.5% and conduct sensitivity as discussed in Note 4.

Note 8: Oceanfront properties are exposed to more risk from storm damage and inundation, and thus there should be a risk premium for oceanfront. We choose 2%, recognizing that a higher (or lower) rate will push oceanfront and non-oceanfront prices closer together (or further apart), as the higher risk premium dissipates the oceanfront amenity premium. Importantly, the base risk premium and depreciation rate are added together in equation 2, so sensitivity analysis can be conducted on either term.

Note 9: Supplemental Figure 6 shows various functional forms for the rise in risk with increasing sea level. The term a_2 sets the value of risk once the property is effectively at the precipice of inundation. The conclusions presented in the main text do not depend sensitively on which particular functional form is chosen. Supplemental Figure 24 shows a comparison of property values for various forms of rising risk. For our baseline simulation we chose a nonlinear increase in risk ($n = 2$) to reflect the likelihood of nonlinear changes in risk as sea level rise increases.

Note 10: The true elevation of property relative to mean sea level on most barrier islands is variable and can certainly be higher than 1m. However, for the purposes of our model, we are interested in a representative elevation between the barrier island and the level of the sea such that when this value is zero, high frequency flooding events become a regular nuisance to portions of the coast. For this we use the sea level rise viewer from NOAA at locations along low lying barrier islands along the East Coast of the U.S.
https://coast.noaa.gov/slr/

Note 11: Storm risks are at least partly capitalized into housing markets. Hallstrom and Smith, 2005[7] estimate a 19% decrease in the appreciation rate for homes in the SFHA of Lee County, Florida after Hurricane Andrew in 1992.

Note 12: The agent risk multiplier captures risk tolerance heterogeneity by allowing risk tolerance to be higher or lower for different agents. At a risk multiplier of one, property values capitalize risk such that they are equal to the expected value of outcomes (equivalent to risk neutrality). A risk multiplier below one implies that the owner is more risk tolerant, whereas a multiplier above one suggests that the owner agent is less risk tolerant.

Note 13: In the range of base property values used in Gopalakrishnan et al., 2011[3]. Chosen specifically to calibrate total property value to the outside market at the beginning of each simulation.

Note 14: Based on empirical estimates and simulations in Gopalakrishnan et al., 2011[3], which best fit the empirical frequency

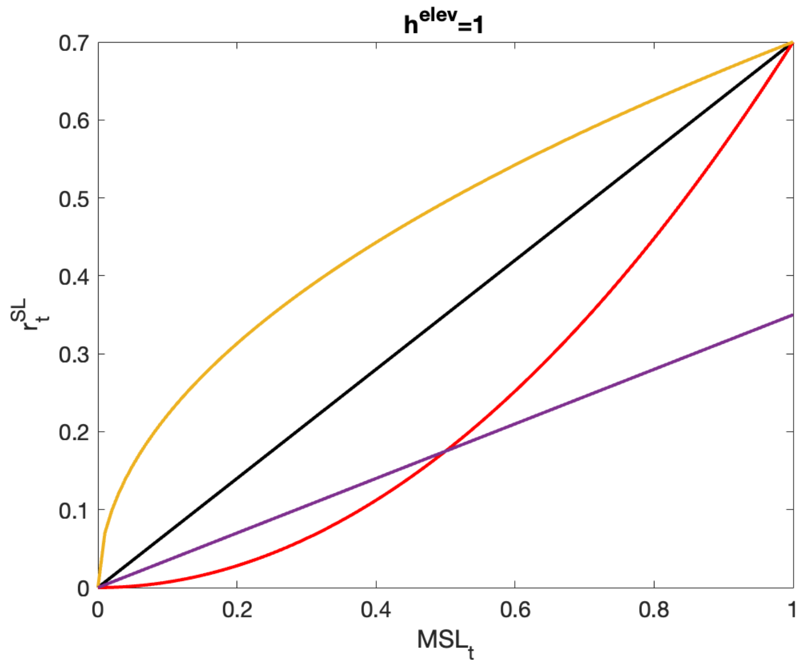

Supplemental Figure 6. Equation 13 from main text. Four forms for the increase in the r_t^{SL} risk parameter versus increasing sea level through time MSL_t. Red, black and yellow correspond to $n = 2, 1, 0.5$ for the SL risk exponent and $a_2 = 0.7$ for the SL risk coefficient. The purple line corresponds to $n = 1$ with $a_2 = 0.35$.

of beach nourishment.

Note 15: Reflects the empirical regularity in the literature[8,9] that beach width has more positive influence on oceanfront properties than on non-oceanfront properties.

Note 16: These hedonic scaling parameters are similar to those in Gopalakrishnan et al., 2011[3]. They also are chosen to calibrate to the outside market at the beginning of each simulation.

Note 17: Federally subsidized projects often plan for 50-year time horizons, whereas non-federal projects can plan for 30-year horizons. We use the shorter time horizon to allow scenarios that reduce or eliminate federal funding.

https://asbpa.org/wp-content/uploads/2020/01/Local-Funding-Report_Final_1.22.20.pdf

Note 18: The goal of this parameter is to have a value that is has comparable amenities to be priced the same as a typical property the simulation. Current (11/20/23) average sales prices in coastal North Carolina include: Wilmington $390,034, Nags Head $699,421, Kill Devil Hills $490,282, Rodanthe $644,493, Wrightsville Beach $1,519,281, Atlantic Beach $521,081, North Topsail Beach $513,282. These figures average across oceanfront/waterfront and non-oceanfront/non-waterfront properties. The current U.S. average is $346,653.

https://www.zillow.com/home-values/

Note 19: Supplemental Figure 7 shows various choices for the bubble coefficient h and the subsequent alteration to the form of Equation 19. The conclusions presented in the main text do not depend sensitively on which particular value is chosen.

Note 20: The value of the flux parameter is chosen so that agents can flux into a market to take advantage of arbitrage opportunities over a time scale of a few years. Increasing this value causes agents to flux in instantly, whereas lower values lead to agents taking many decades to flux into a market. See additional discussion below and Supplemental Figure 9.

Note 21: There is considerable heterogeneity in tax ratios for special municipal service districts to fund beach nourishment.

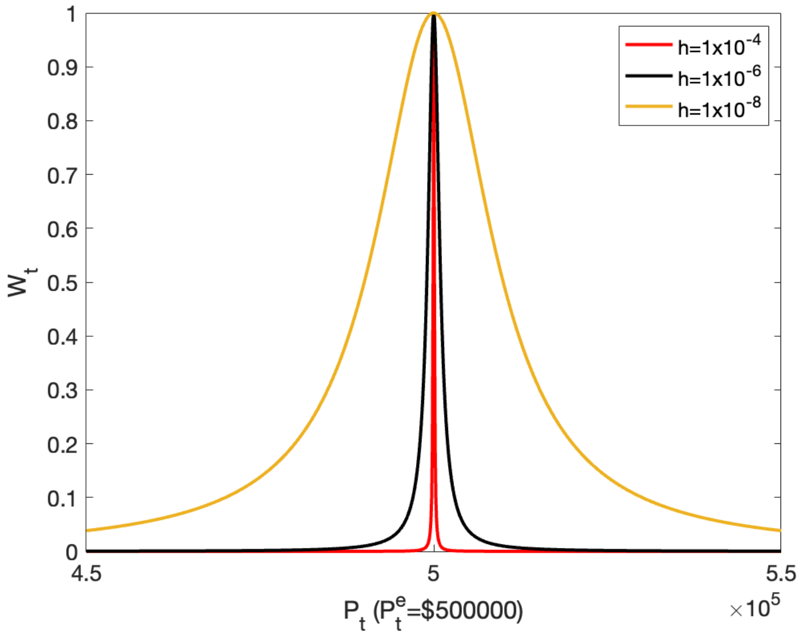

Supplemental Figure 7. Equation 19 from main text. Three values of the bubble coefficient and their impact on changing W_t versus the value of property in a market P_t, where the outside equilibrium price P_t^e is set to $500,000.

Some municipalities do not use this mechanism, which leads implicitly to a ratio of 1:1. Others more aggressively tax oceanfront properties, with ratios as high as 10-15[10,11]. The choice of 3 is in the middle of these extreme ranges.

Note 22: When taken together these terms from the simulated nourishment events are within range of the total cost of typical beach nourishment projects along the East Coast of the Untied States and are similar to cost parameters in the literature[12,13].

Note 23: https://oceanservice.noaa.gov/hazards/sealevelrise/sealevelrise-tech-report.html

3 Rationale for Scenarios

Justification of the Baseline Case – 90% Nourishment Subsidy

The 90% baseline captures the typical case. Although there is considerable variation in how beach nourishment projects are funded, local funding from property taxes typically constitutes a small share of the total. For most beach nourishment projects, the federal subsidy has been approximately two thirds of the cost with the remaining one third financed by a combination of indirect federal subsidies for inlet stabilization and dredge disposal, state contributions, hotel taxes, local sales taxes, and local property taxes[14–17]. Local property taxes are the non-subsidized component in the sense that it is paid directly by property owner beneficiaries of the project. Some projects are cost-shared between state, federal, and local funding with others having federal and state cost sharing that covers the entire cost of the project, e.g. in Louisiana[18]. In some places, such as Kure Beach, NC, local property taxes do not pay any share of the project[19]. Federal, state, and local funding shares vary substantially across projects in South Carolina with local funding providing no contributions in many instances but shouldering the entire burden in others[20]. Historically, between 65% and 85% of beach nourishment projects have had a federal component[21]. In Figure 4 of Valverde, Trembanis, and Pilkey (1999), 43% is federal storm and erosion, 14% is federal navigation, 6% is federal emergency, 2% is state, 18% is state and local cooperative agreements, and only 9% of funding is classified as local/private with 8% as unknown.

Justification for Scenario 1 – Nourishment Subsidy Cut from 90% to 50%

The reduction to a 50% subsidy is a plausible change based on the political economy over the past two decades during which there has been momentum to reduce the federal share of funding for beach nourishment dramatically, decrease state contributions, and increase the share shouldered by local sources. Both the William Clinton and George W. Bush Administrations proposed cutting the two-thirds federal share in half, although Congress maintained subsidies at a higher level during their administrations[22,23]. Cutting the federal share in half alone would reduce the total subsidy to 57%, and there appears to be a similar push to force local communities to shoulder a greater share at the state level[10,17].

Justification for Scenario 2 - Appreciation in outside real estate markets, namely a doubling in 50 years.

This scenario is based on historic real (inflation-adjusted) appreciation in national real estate markets in the United States and projecting that this appreciation will continue into the future. Specifically, doubling real prices in 50 years is a conservative projection of continued real estate appreciation from 1987 to the present (the period for which a consistent national real estate index is available). Doubling in 50 years implies a 1.4% appreciation rate. The historical real rate of appreciation in U.S. real estate markets (after adjusting for inflation) is 1.6%. This rate is calculated from the S&P/Case-Shiller U.S. National Home Price Index, Index Jan 2000=100, Monthly, Seasonally Adjusted, which is converted from nominal to real using the Consumer Price Index for All Urban Consumers: All Items in U.S. City Average, Index 1982-1984=100, Monthly, Seasonally Adjusted. The resulting 1.6% is the compounded real rate of appreciation over the period spanning January 1987 through July 2023.

Justification for Scenario 3 – Constant outside real estate markets and then dramatic decline, namely 90% depreciation in 50 years.

This scenario is exploratory in nature because real estate markets are not guaranteed to appreciate and could depreciate. We chose a substantial long-term depreciation rate to explore specifically if the demographic changes predicted by the model could be reversed. That said, this real rate of depreciation is not outside the rate of depreciation experienced recently on the decadal scale in housing markets. A 90% deprecation over 50 years corresponds to an annual rate of 4.5% depreciation. Based on the S&P/Case-Shiller U.S. National Home Price Index discussed above, between 2006 and 2012, housing markets depreciated at an annual rate of 7%, which is considerably faster than the 4.5% implied by our model.

4 Sensitivity Analysis

Random Draws: The model endogenously updates the parameters of agent distributions, and in each time step agent parameters are drawn randomly. Because there are a number of sources of nonlinearities and the potential for these nonlinearities to propagate over time, we explore the robustness of the baseline model to different sets of random draws. To this end, we repeatedly change the seed for the random number generator and re-run the baseline model. We use the resulting set of simulations to trace out 95% confidence intervals on the simulations. Supplemental Figure 8 shows the results. For most variables, the resulting confidence intervals are quite narrow. The main conclusions of the model are unchanged.

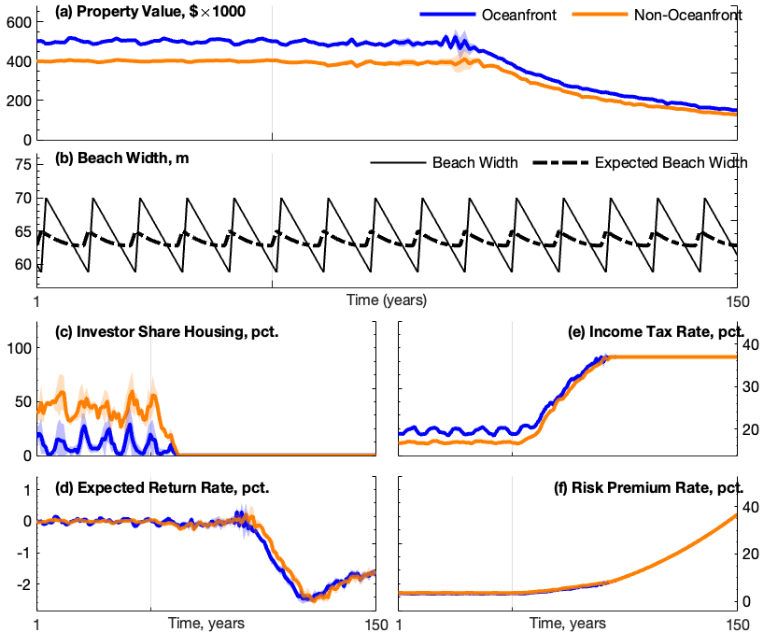

Supplemental Figure 8. Baseline simulation and results as in Figure 3 from the main text, here with shaded areas showing the 95% confidence intervals.

Parameter Values: The strength of empirical foundation varies for each parameter in the model, and some parameters are expected to have more influence on model outcomes than others. Based on these factors, we conduct sensitivity for a number of parameters by positing a low value (below the value used in the main text) and a high value (above the value used in the main text) and re-running the model for each value. For some parameter sensitivity analyses, we re-run the baseline model and compare to the original baseline as a means to understand how the mechanisms are working. For others, we re-run one of the policy experiments, namely the cut to the beach nourishment subsidy, using the lower or higher parameter value in both the baseline and the intervention. For two cases, namely the discount rate and the base property value parameter, we do both. The parameter value ranges are discussed in more detail in Supplemental Table 1.

Flux parameter (ϕ) – To examine to the sensitivity of the model to the flux parameter, we highlight the speed of adjustment under two flux values in Supplemental Figure 9. The first (4x baseline) leads to rapid adjustment in which the property value adjusts almost instantaneously to outside markets (within 2 years). The second (0.1x baseline) is a slow adjustment that unfolds over 30 years. These two extremes—unrealistically short or long speed of adjustment—provide justification of our baseline flux parameter choice.

Hedonic Scaling (α) – To capture regional variation in property values independent of climate risk and beach width, we conduct sensitivity on the base home value. First, we show how these changes do not affect the qualitative conclusions of the nourishment policy experiment. Second, we compare each case to the baseline to ensure that the mechanisms are working as we expect.

In the low alpha case, results are qualitatively the same as in the main text (Supplemental Figure 10). The difference is that property values can be sustained for longer because fewer high-income owners have entered the market at the onset of SLR. In the high alpha case, results are similar in that the subsidy only temporarily maintains property values in the face of SLR, but

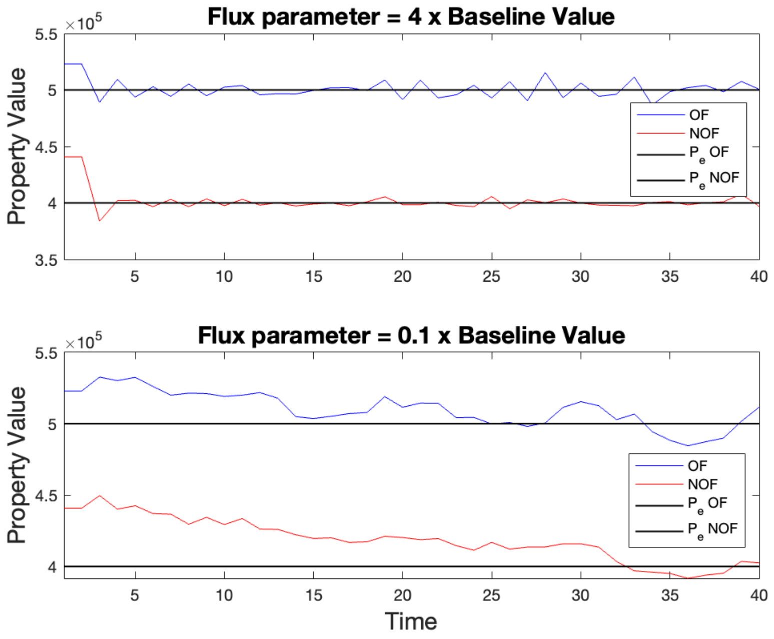

Supplemental Figure 9. Oceanfront (OF) and Non oceanfront (NOF) property values though time for two flux parameter values indicated. In both simulations the values are relaxing to the equilibrium values (black) due to the flux of agents into or out of the market.

there is a shorter period in which high-income owners can enter before saturating the market (Supplemental Figure 11). At first, the pattern of nourishment remains the same compared to our original policy experiment. However, after roughly 30 years, an additional nourishment cycle begins, which the higher base property value justifies. So, we see some recovery in property values and beach width relative to the baseline 90% subsidy case.

When we compare to the baseline, we can clearly see how the mechanism is working (Supplemental Figure 12 and 13). Before SLR, the lower base property decreases property values, and the higher base property increases property values. Lower base property value decreases the average tax rate, and higher base property value increases the average tax rate. With lower alpha, the demographic shift narrows the gap in property value compared to the baseline, whereas with higher alpha, the demographic shift exacerbates the gap. Higher SLR just erodes property values overall relative to the baseline with lower SLR.

Discount rate (δ) – The discount rate is often a key driver of outcomes in dynamic economic analysis. In the user cost model, it interacts with the marginal tax rate and, as such, affects property values in ways that endogenously depend on incomes of owners. We re-run the nourishment policy experiment using a low discount rate (3%) and a high discount rate (9%). Compared to our findings in the main text, nothing changes qualitatively. We see that the decline in property value begins later for the low discount rate case. This timing difference is due to the fact that, with the lower discount rate, there is more room in the market for high-income owners to flux in (because they are less tax advantaged compared to the cases with higher discount rates).

To ensure that the mechanism is working as we expect, we also run the low and high discount rate cases relative to the baseline 6% discount case. Here we see clearly that, with the low discount rate, property values are slightly higher before the onset of SLR and more so for non-oceanfront because they are taxed less to fund nourishment. Before SLR, owners are lower-income relative to the baseline because the lower discount rate creates less tax advantage (recall discount rate is multiplied by marginal tax rate). After the onset of SLR, wealthier owners flux in and drive prices up further. The opposite is true in the high discount rate case – lower prices due to less capitalization and more tax advantage for high-income owners so fewer high-income owners who can flux in later. Results of the discount rate sensitivity analysis appear in Supplemental Figures 14,15, 16,17.

Depreciation (γ) – Conducting sensitivity on depreciation is equivalent to conducting sensitivity on baseline risk premium of residential real estate because the parameters are summed in the denominator of the user cost equation. Supplemental Figures 18 and 19 show the results.

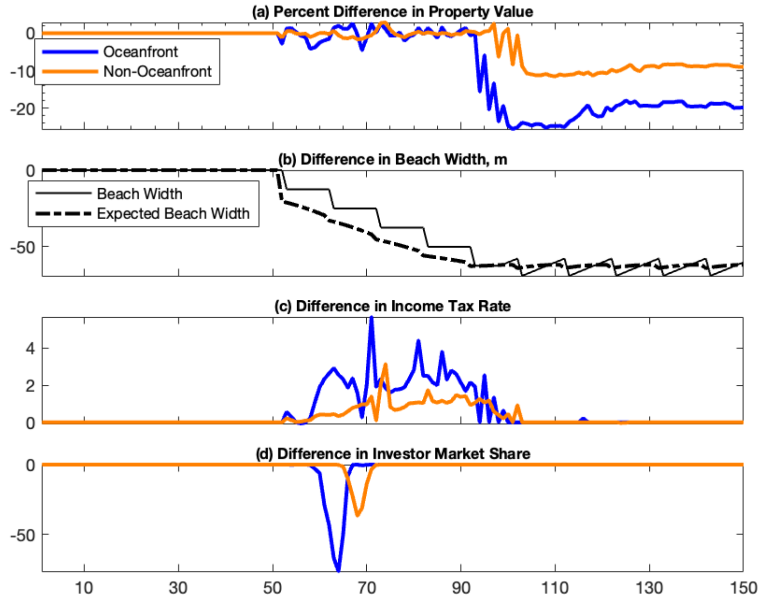

Supplemental Figure 10. Model results for scenario where the baseline hedonic parameter range has been decreased by 20% and the nourishment subsidy is turned off at year 50. Results displayed as differences compared to the baseline nourishment subsidy removal scenario.]

Oceanfront Risk (r^{OF}) – Oceanfront properties are exposed to more risk from storm damage and inundation, and thus there should be a risk premium for oceanfront. We vary the oceanfront risk premium from 0.01 to 0.03. Supplemental Figures 20 and 21 show the results.

Summary We can also look at the sensitivity analyses together for the subset of parameters that enter the user cost model directly. The following heatmaps (Supplemental Figures 22 and 23) combine these parameter sensitivity analyses to look at the effect on property values. These figures reinforce the finding that the parameter differences can alter the timing of property value declines but not the main result that the mechanisms in the model only serve to delay inevitable property value declines.

Functional Form: As described in Supplemental Table 1, the parameters n and a_2 control the shape and magnitude of the risk from sea-level rise. Different levels of n effectively accelerate or delay the large effects of SLR on property values. The results in Supplemental Figure 24 show that, not surprisingly, property values adjust downward more slowly when n is high because the effect of SLR on risk is worsening over time. When n is low, the opposite is the case because the effect of SLR on risk is strongest at the onset of SLR. The lower parameter a_2 corresponds to a smaller magnitude of risk and thus a higher ending property value as the system adjusts.

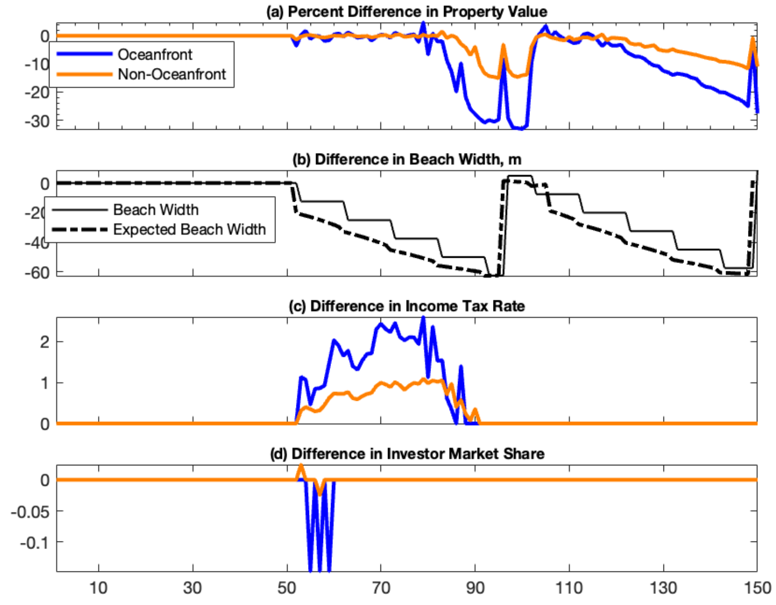

Supplemental Figure 11. Model results for scenario where the baseline hedonic parameter range has been increased by 20% and the nourishment subsidy is turned off at year 50. Results displayed as differences compared to the baseline nourishment subsidy removal scenario.]

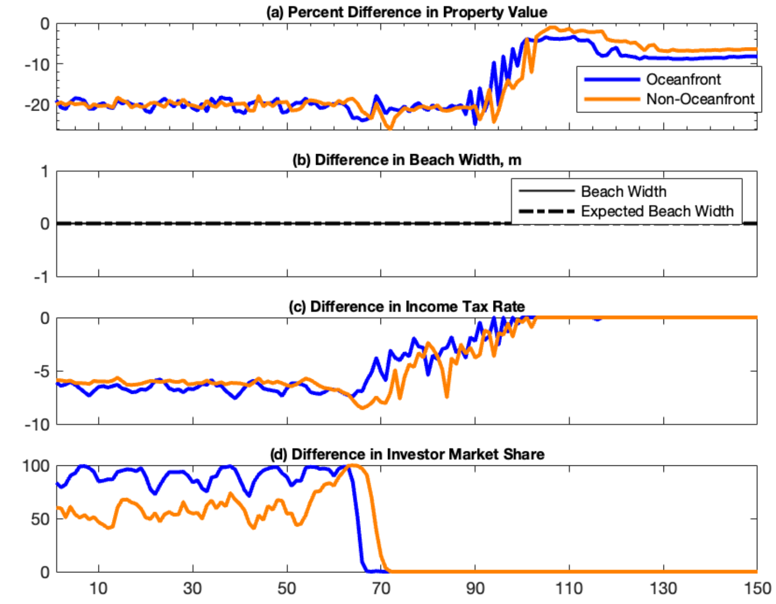

Supplemental Figure 12. Model results for scenario where the baseline hedonic parameter range has been decreased by 20%. Results displayed as differences compared to the baseline scenario.

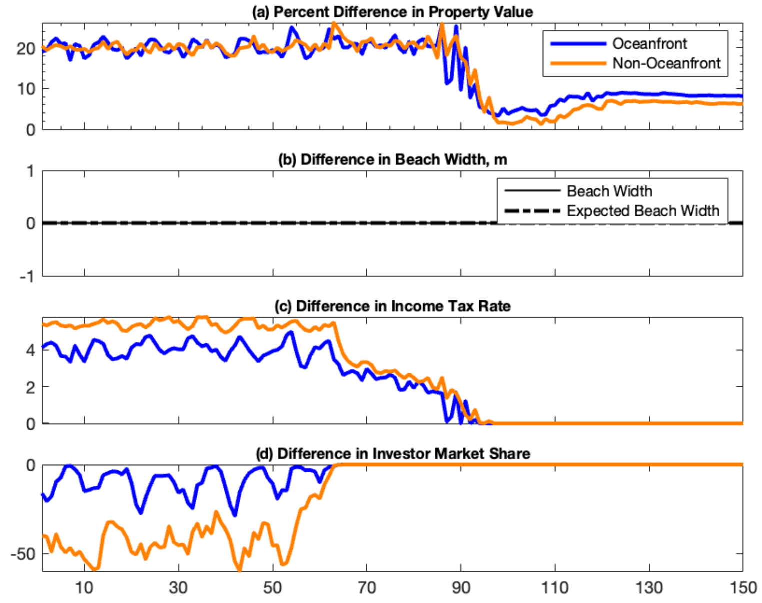

Supplemental Figure 13. Model results for scenario where the baseline hedonic parameter range has been increased by 20%. Results displayed as differences compared to the baseline scenario.

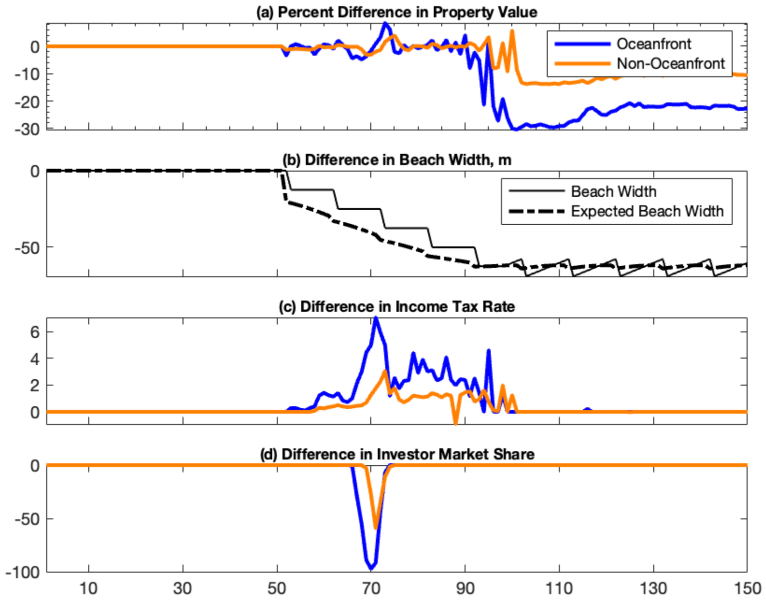

Supplemental Figure 14. Model results for scenario where the baseline discount rate parameter range has been decreased to 3% and the nourishment subsidy is reduced at year 50. Results displayed as differences compared to the baseline nourishment subsidy removal scenario.

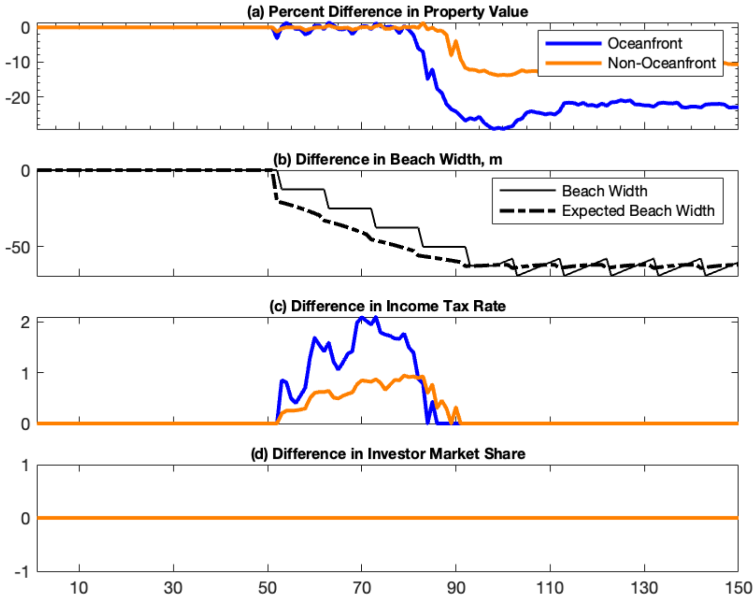

Supplemental Figure 15. Model results for scenario where the baseline discount rate parameter range has been increased to 9% and the nourishment subsidy is reduced at year 50. Results displayed as differences compared to the baseline nourishment subsidy removal scenario.

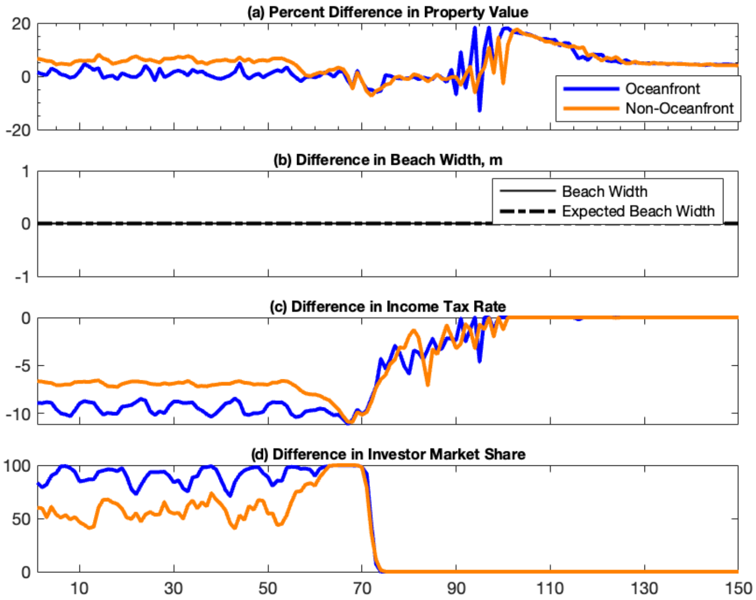

Supplemental Figure 16. Model results for scenario where the baseline discount rate parameter has been decreased to 3%. Results displayed as differences compared to the baseline scenario.

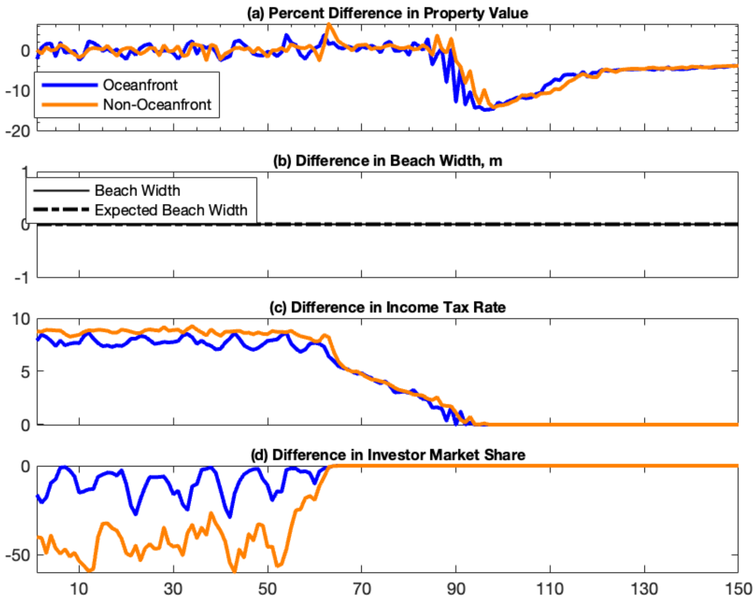

Supplemental Figure 17. Model results for scenario where the baseline discount rate parameter has been increased to 9%. Results displayed as differences compared to the baseline scenario.

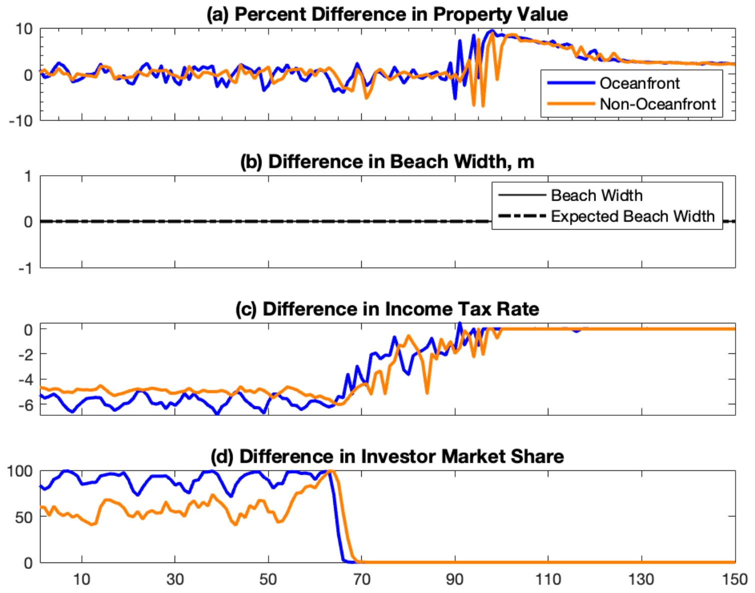

Supplemental Figure 18. Model results for scenario where the baseline depreciation rate parameter has been decreased to 0%. Results displayed as differences compared to the baseline scenario.

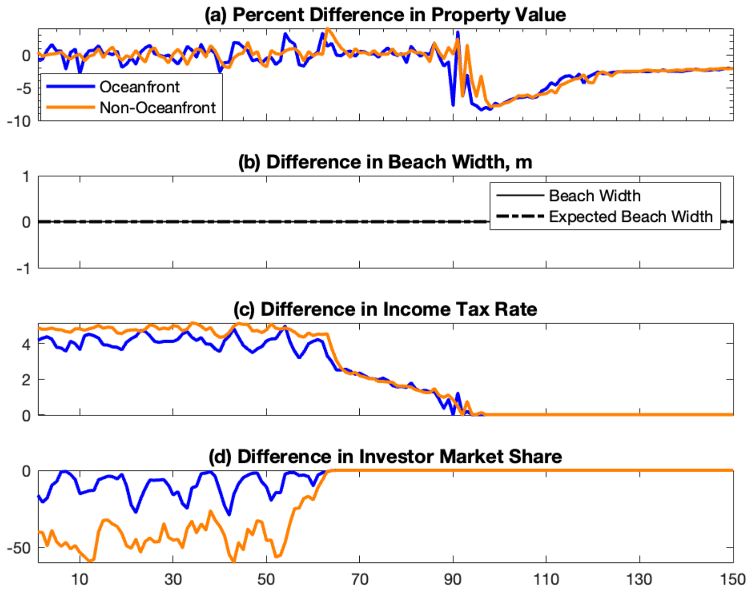

Supplemental Figure 19. Model results for scenario where the baseline depreciation rate parameter has been increased to 2%. Results displayed as differences compared to the baseline scenario.

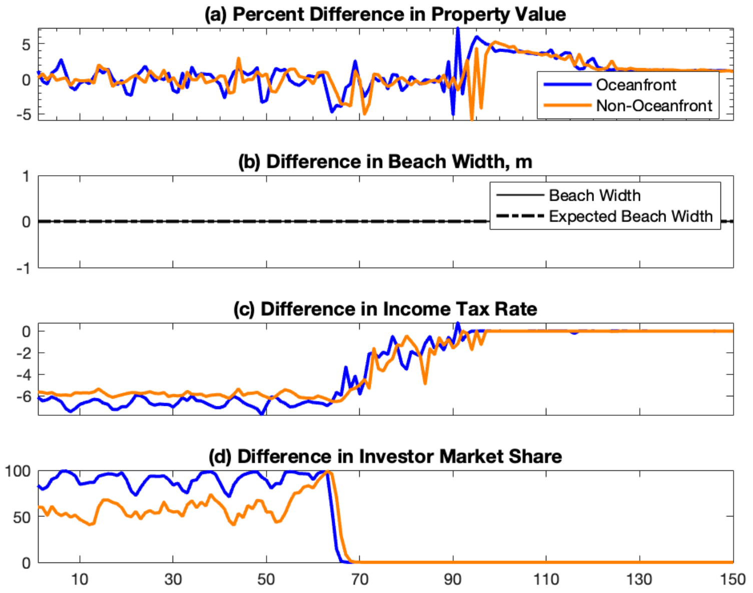

Supplemental Figure 20. Model results for scenario where the baseline oceanfront risk parameter has been decreased to 1%. Results displayed as differences compared to the baseline scenario.

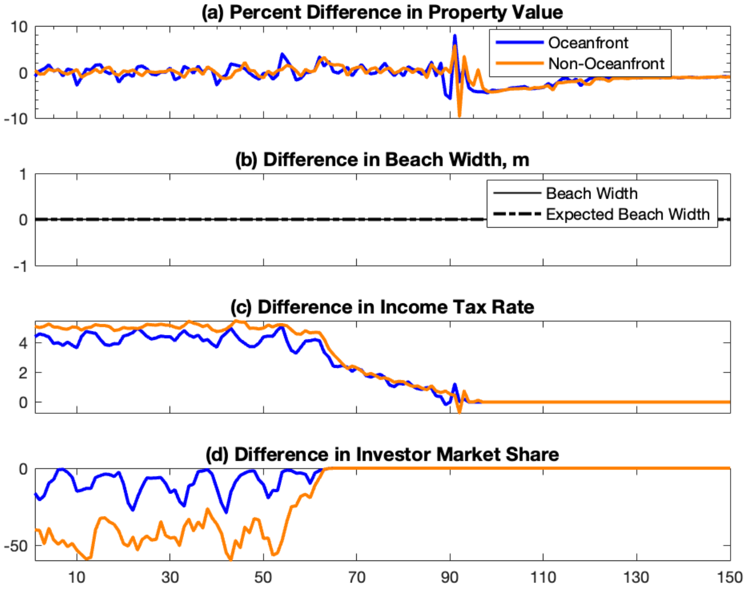

Supplemental Figure 21. Model results for scenario where the baseline oceanfront risk parameter has been increased to 3%. Results displayed as differences compared to the baseline scenario.

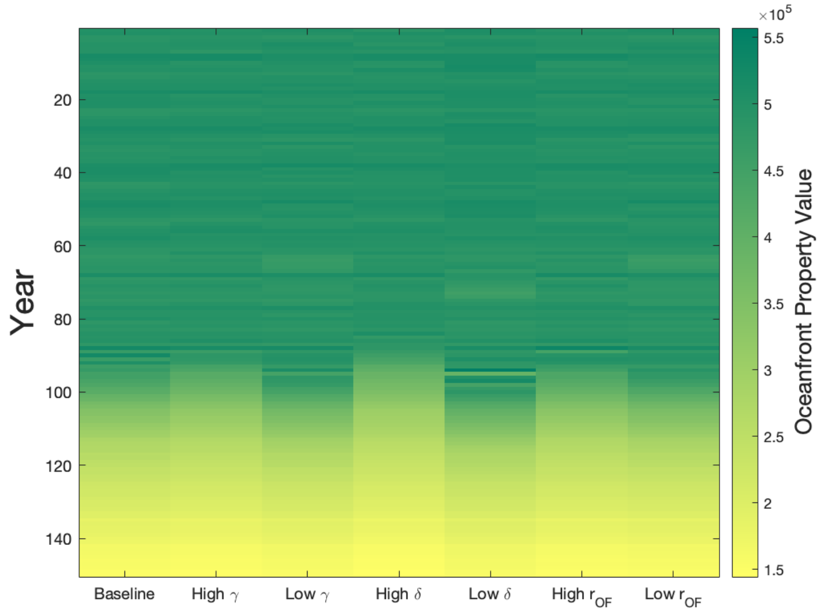

Supplemental Figure 22. Model results for sensitivity scenarios as noted on the horizontal axis. Oceanfront property value is shown in colors with simulation year indicated on the vertical axis.

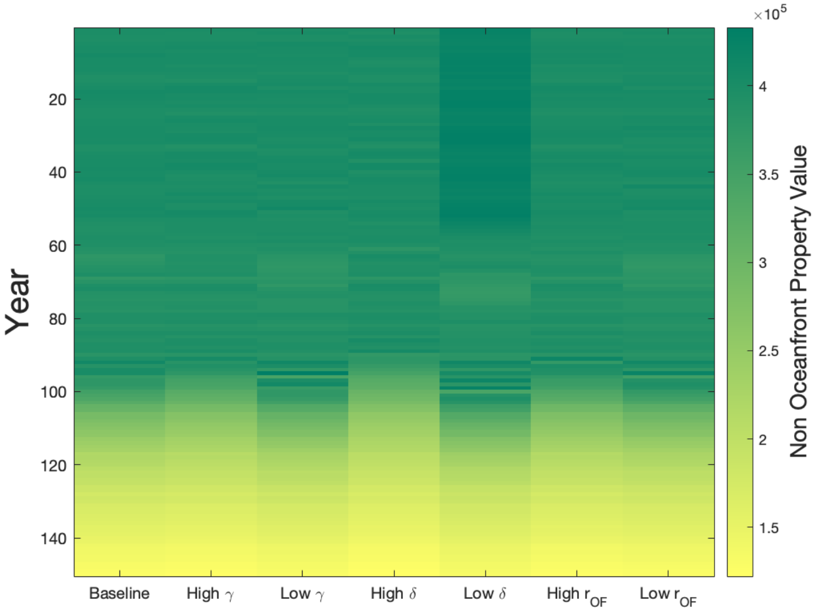

Supplemental Figure 23. Model results for sensitivity scenarios as noted on the horizontal axis. Non oceanfront property value is shown in colors with simulation year indicated on the vertical axis.

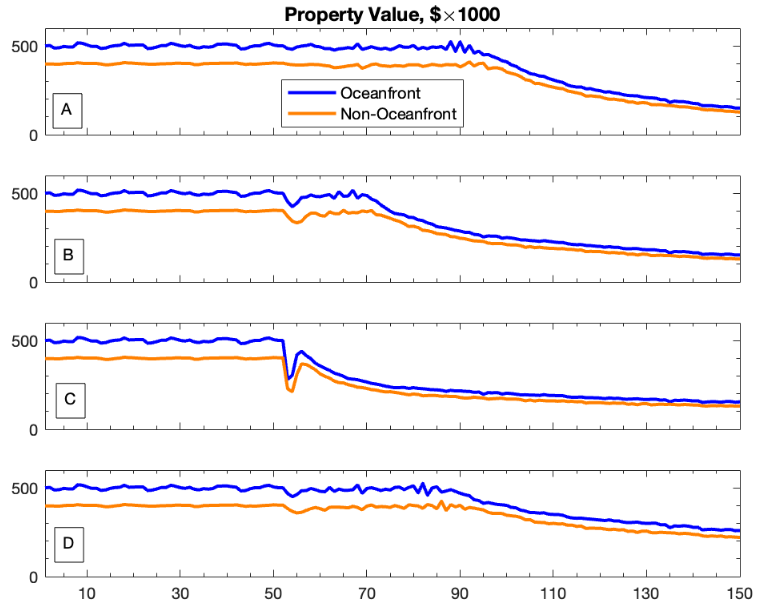

Supplemental Figure 24. Property values for different risk forms. The time series of property value in a baseline model setting (as described in the main text) for the four different forms for risk shown in Supplemental Figure 6. A, B, C, D correspond to risk as set in the red, black, yellow, and purple plots from that figure.

5 Additional Scenarios

Scenario 2 We consider two alternatives to our reduced nourishment subsidy scenario. In the main text, the subsidy is reduced from 90% to 50%. Here we consider reducing the subsidy to 25% or 75%.

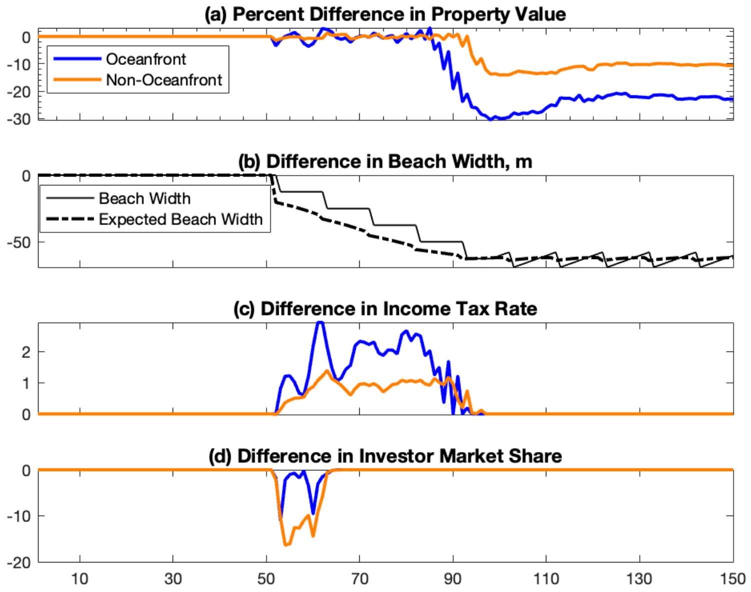

Supplemental Figure 25. Model results for scenario where the nourishment subsidy is turned down to 25% at year 50. Results displayed as differences compared to the baseline nourishment subsidy removal scenario.

Scenario 3 We consider two alternative scenarios to increasing outside real estate markets. Specifically, we consider outside markets that appreciate to 1.5 times the starting value over 50 years (compared to doubling in the main text scenario) and markets that appreciate to 2.5 times the starting value over 50 years.

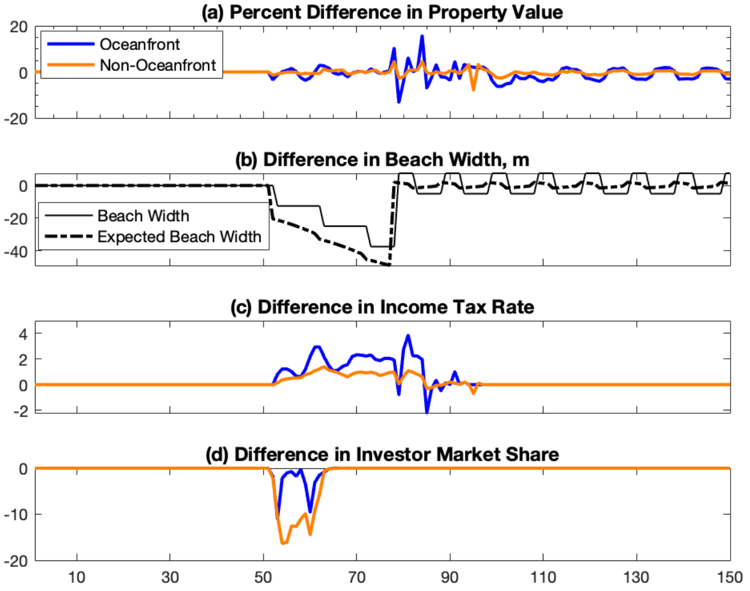

Supplemental Figure 26. Model results for scenario where the nourishment subsidy is turned down to 75% at year 50. Results displayed as differences compared to the baseline nourishment subsidy removal scenario.

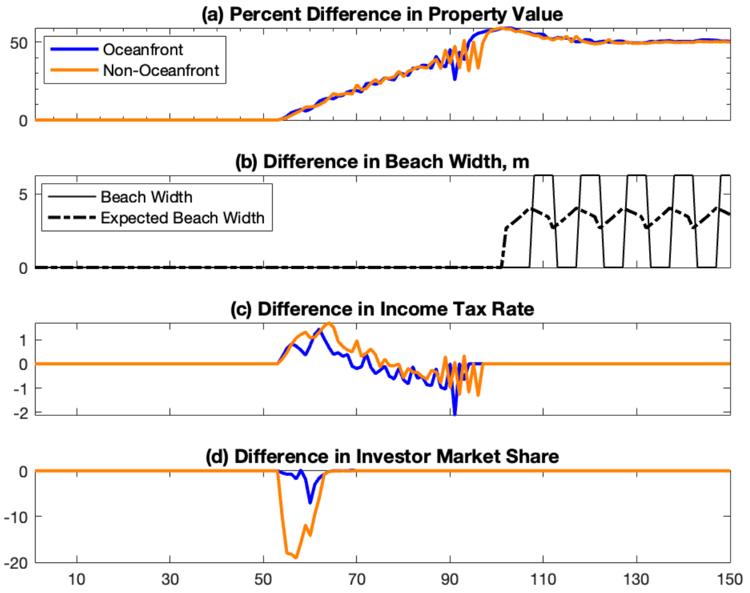

Supplemental Figure 27. Model results for scenario where the outside market appreciate by 50% over 50 years. Results displayed as differences compared to the baseline outside market appreciation scenario.

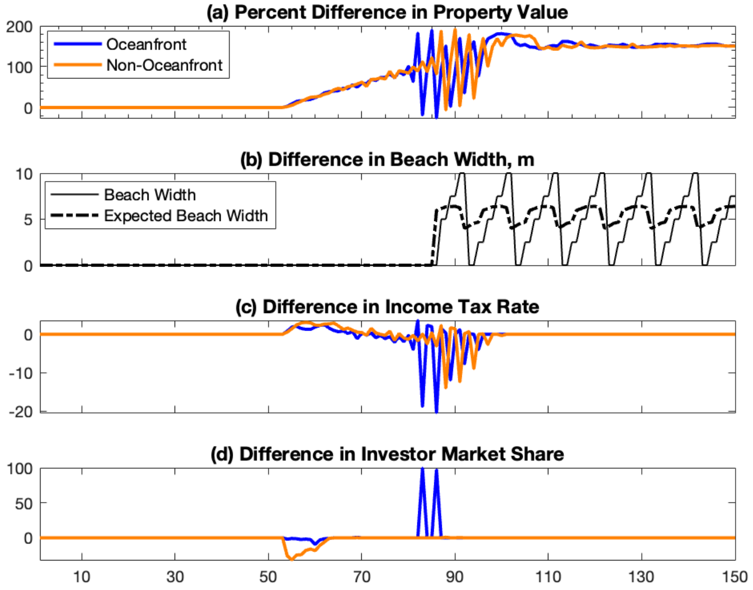

Supplemental Figure 28. Model results for scenario where the outside market appreciate by 150% over 50 years. Results displayed as differences compared to the baseline outside market appreciation scenario.

References

1. Keim, B. D., Muller, R. A. & Stone, G. W. Spatiotemporal patterns and return periods of tropical storm and hurricane strikes from texas to maine. *J. Clim.* **20**, 3498–3509 (2007).
2. Hapke, C. J., Himmelstoss, E. A., Kratzmann, M. G. & Thieler, E. R. National assessment of shoreline change: Historical shoreline change along the new england and mid-atlantic coasts: Technical report, u.s.g.s open-file report 2010-1118. (2011).
3. Gopalakrishnan, S., Smith, M. D., Slott, J. M. & Murray, A. B. The value of disappearing beaches: a hedonic pricing model with endogenous beach width. *J. Environ. Econ. Manag.* **61**, 297–310 (2011).
4. Malpezzi, S., Ozanne, L. & Thibodeau, T. G. Microeconomic estimates of housing depreciation. *Land Econ.* **63**, 372–385 (1987).
5. Harding, J. P., Rosenthal, S. S. & Sirmans, C. Depreciation of housing capital, maintenance, and house price inflation: Estimates from a repeat sales model. *J. Urban Econ.* **61**, 193–217 (2007).
6. Cannon, S., Miller, N. G. & Pandher, G. S. Risk and return in the us housing market: A cross-sectional asset-pricing approach. *Real Estate Econ.* **34**, 519–552 (2006).
7. Hallstrom, D. G. & Smith, V. K. Market responses to hurricanes. *J. Environ. Econ. Manag.* **50**, 541–561 (2005).
8. Hazard, F. Coastal housing market response to amenities and risk,”. *Land Econ.* .
9. Landry, C. E., Turner, D. & Allen, T. Hedonic property prices and coastal beach width. *Appl. Econ. Perspectives Policy* **44**, 1373–1392 (2022).
10. Mullin, M., Smith, M. D. & McNamara, D. E. Paying to save the beach: effects of local finance decisions on coastal management. *Clim. Chang.* **152**, 275–289 (2019).
11. Thompson, A. Rising seas, falling funds: An analysis of beach nourishment finance in dare county, nc. (2018).
12. Smith, M. D., Slott, J. M., McNamara, D. & Murray, A. B. Beach nourishment as a dynamic capital accumulation problem. *J. Environ. Econ. Manag.* **58**, 58–71 (2009).
13. McNamara, D. E., Murray, A. B. & Smith, M. D. Coastal sustainability depends on how economic and coastline responses to climate change affect each other. *Geophys. Res. Lett.* **38** (2011).
14. North carolina DENR. 2011. NC Beach and Inlet Management Plan – Final Report. https://www.deq.nc.gov/documents/pdf/bimp/bimp-section-xii-funding-prioritization-formatted/download. Accessed: 2023-12-10.
15. Brockbank, D. *et al.* Local funding for coastal projects: An overview of practices, policies, and considerations. *Shore & Beach* **88**, 53 (2020).
16. Gopalakrishnan, S., Landry, C. E. & Smith, M. D. Climate change adaptation in coastal environments: modeling challenges for resource and environmental economists. *Rev. environmental economics policy* (2018).
17. Star News. https://www.starnewsonline.com/story/news/2021/08/13/north-carolina-beach-maintenance-costs-rising-amid-climate-change 5567860001/. Accessed: 2023-12-10.
18. Elko, N. *et al.* A century of us beach nourishment. *Ocean. & Coast. Manag.* **199**, 105406 (2021).
19. Kure Beach. https://www.townofkurebeach.org/sites/default/files/uploads/beach-nourishment-_rot-brochure-2019.pdf. Accessed: 2023-12-10.
20. Houston, J. R. The economic value of beach nourishment in south carolina. *Shore Beach* **89**, 3–12 (2021).
21. Trembanis, A. C., Pilkey, O. H. & Valverde, H. R. Comparison of beach nourishment along the us atlantic, great lakes, gulf of mexico, and new england shorelines. *Coast. Manag.* **27**, 329–340 (1999).
22. Tampa Bay. https://www.tampabay.com/archive/1999/07/06/costly-beach-proposal-resisted/.
23. New Jersey. https://archive.centraljersey.com/2001/07/05/beach-replenishment-still-a-federal-project-presidents-plan-to-reduce-funding-overturned-in-the-house-of-representatives/.
