# Supplementary material for: Policy and market forces delay real estate price declines on the US coast
Source: Nat Commun. 2024 Mar 12;15:2209. doi: 10.1038/s41467-024-46548-6 (PMC10928214; doi:10.1038/s41467-024-46548-6)
Supplement: Supplementary file 3 — Reporting Summary [file 41467_2024_46548_MOESM3_ESM.pdf]

nature portfolio

Corresponding author(s): Martin D. Smith

Last updated by author(s): Jan 24, 2024

Reporting Summary

Nature Portfolio wishes to improve the reproducibility of the work that we publish. This form provides structure for consistency and transparency in reporting. For further information on Nature Portfolio policies, see our Editorial Policies and the Editorial Policy Checklist.

Statistics

For all statistical analyses, confirm that the following items are present in the figure legend, table legend, main text, or Methods section.

n/a	Confirmed	
☐	☒	The exact sample size (*n*) for each experimental group/condition, given as a discrete number and unit of measurement
☒	☐	A statement on whether measurements were taken from distinct samples or whether the same sample was measured repeatedly
☒	☐	The statistical test(s) used AND whether they are one- or two-sided<br>*Only common tests should be described solely by name; describe more complex techniques in the Methods section.*
☐	☒	A description of all covariates tested
☐	☒	A description of any assumptions or corrections, such as tests of normality and adjustment for multiple comparisons
☒	☐	A full description of the statistical parameters including central tendency (e.g. means) or other basic estimates (e.g. regression coefficient) AND variation (e.g. standard deviation) or associated estimates of uncertainty (e.g. confidence intervals)
☒	☐	For null hypothesis testing, the test statistic (e.g. *F*, *t*, *r*) with confidence intervals, effect sizes, degrees of freedom and *P* value noted<br>*Give P values as exact values whenever suitable.*
☒	☐	For Bayesian analysis, information on the choice of priors and Markov chain Monte Carlo settings
☒	☐	For hierarchical and complex designs, identification of the appropriate level for tests and full reporting of outcomes
☒	☐	Estimates of effect sizes (e.g. Cohen's *d*, Pearson's *r*), indicating how they were calculated

Our web collection on statistics for biologists contains articles on many of the points above.

Software and code

Policy information about availability of computer code

Data collection

No software was used for data collection.

Data analysis

Model simulations are run in Matlab version R2022b. The Matlab code can be accessed at https://github.com/818 dylanmcn/C HOM. Figure 1 in the Introduction was drawn in Stata version 18.0, and the code that ran the underlying regression model and averages the county-year effects is deposited in https://github.com/dylanmcn/C HOM.

For manuscripts utilizing custom algorithms or software that are central to the research but not yet described in published literature, software must be made available to editors and reviewers. We strongly encourage code deposition in a community repository (e.g. GitHub). See the Nature Portfolio guidelines for submitting code & software for further information.

Data

Policy information about availability of data

All manuscripts must include a data availability statement. This statement should provide the following information, where applicable:

- Accession codes, unique identifiers, or web links for publicly available datasets
- A description of any restrictions on data availability
- For clinical datasets or third party data, please ensure that the statement adheres to our policy

The data used to support our findings are the outputs of numerical model simulations. The model is documented in the Methods section of the paper, and the

parameterization is documented in detail in the Supplemental Material.

The data underlying Figure 1, not part of results but motivating the study, are from a nationwide dataset with 23,184,659 sales transaction records acquired from a private data provider CoreLogic Inc. CoreLogic provides proprietary real estate data with sales prices, housing attributes (including square footage, number of bedrooms and bathrooms, lot size, construction type, and the presence of a pool), and location information. (https://www.corelogic.com)
County-by-Year Price Indices are estimated for 665 counties from 1989 to 2016 in the states along the US Atlantic and Gulf Coast

Research involving human participants, their data, or biological material

Policy information about studies with human participants or human data. See also policy information about sex, gender (identity/presentation), and sexual orientation and race, ethnicity and racism.

Reporting on sex and gender	n/a
Reporting on race, ethnicity, or other socially relevant groupings	n/a
Population characteristics	n/a
Recruitment	n/a
Ethics oversight	n/a

Note that full information on the approval of the study protocol must also be provided in the manuscript.

Field-specific reporting

Please select the one below that is the best fit for your research. If you are not sure, read the appropriate sections before making your selection.

☐ Life sciences ☒ Behavioural & social sciences ☐ Ecological, evolutionary & environmental sciences

For a reference copy of the document with all sections, see nature.com/documents/nr-reporting-summary-flat.pdf

Behavioural & social sciences study design

All studies must disclose on these points even when the disclosure is negative.

Study description	This study uses a quantitative numerical model simulation. The study design and numerical experiments are used to investigate mechanisms in the coupled human-natural system that give rise to surprising phenomena documented empirically elsewhere in the literature.
Research sample	No original data were collected for this study. The model was parameterized using published studies in the peer-reviewed and gray literature, websites of government agencies, and secondary data as documented in the Supplemental Materials.
Sampling strategy	No sampling was done. The model output reports a complete census of our results. The motivating Figure 1 uses all sales from 1989 to 2016 along the East and Gulf Coasts of the US.
Data collection	No original data were collected for this study.
Timing	Model simulations include 50 years prior to the onset of SLR and 100 subsequent years. The sales transactions underlying Figure 1 include 1989 through 2016.
Data exclusions	No data were excluded from the analyses.
Non-participation	No participants were involved in this study.
Randomization	Randomization of treatment and control is not relevant to this study because "treatment" is a numerical experiment that turns on or off mechanisms in the numerical model.

Reporting for specific materials, systems and methods

We require information from authors about some types of materials, experimental systems and methods used in many studies. Here, indicate whether each material, system or method listed is relevant to your study. If you are not sure if a list item applies to your research, read the appropriate section before selecting a response.

Materials & experimental systems

n/a	Involved in the study
☒	☐ Antibodies
☒	☐ Eukaryotic cell lines
☒	☐ Palaeontology and archaeology
☒	☐ Animals and other organisms
☒	☐ Clinical data
☒	☐ Dual use research of concern
☒	☐ Plants

Methods

n/a	Involved in the study
☒	☐ ChIP-seq
☒	☐ Flow cytometry
☒	☐ MRI-based neuroimaging

Plants

Seed stocks	n/a
Novel plant genotypes	n/a
Authentication	n/a
